# A Manual of Modern Surgery

**Published:** 1891-02

**Authors:** 


A Manual of Modern Surgery. An Exposition of the Accepted Doctrines and Approved Operative Procedures of the Present Time. For the use of students and practitioners. By John B. Roberts, A. M., M. D., Professor of Surgery in the Woman's Medical College of Pennsylvania; Professor of Anatomy and Surgery in the Philadelphia Polyclinic; Lecturer in Anatomy in the University of Pennsylvania. Octavo, 780 pages, 501 illustrations. Cloth, $4.50; leather, $5.50. Philadelphia: Lea Brothers & Co. 1890.

The name of the author would lead us to expect something quite above the average, and the reader of this Manual of Modern Surgery will have his expectations completely realized. In this work we find the same impartial and common sense method of dealing with questions which have characterized his previous writings. In the preface, he says, "the opinions of the best authorities, the methods of the most practical surgeons, and the well-established facts of surgical science are discussed; but the consideration of theories, historical questions, traditional views and operations and innovations of undecided value, has been rigidly avoided."

In all this choosing and rejecting the author has shown excellent judgment, so that the reader has nothing but strictly modern and accepted surgical principles and practice to occupy his attention. Clearness and conciseness are conspicuous throughout the entire work. Personality, too, is stamped upon every page.

The author begins his work with the consideration of inflammation, then takes up destructive inflammatory processes, such as suppuration, ulceration, gangrene, etc., then general surgical diseases like erysipelas, pyemia, tuberculosis, syphilis, rachitis and others are discussed from the standpoint of a practical surgeon.

In the chapter upon Plastic or Reparative Surgery, which occupies only about seven pages, the author has given us the salient points regarding this special field of surgery.

Part II. is devoted to the surgical diseases of the various tissues and regions of the body. One of the best chapters in this part is that upon the Nerve-centres and Nerves. The author would substitute contusion or laceration for the term concussion of the brain, and he regards the set of symptoms which we call compression of the brain, as due to concomitant inflammation as well as actual compression from extravasated blood, depressed bone, etc. The treatment of cranial fractures, although occurring in another part of the book, may be mentioned here. The author is more radical than most surgeons would deem justifiable. A fairly large experience with this class of cases leads us to believe him much nearer right than wrong. We have never regretted working along the lines he has indicated; on the contrary we have upon two occasions regretted not doing so. An admirable syllabus of treatment of cranial fractures is given, in which the author's views are contained "in a nutshell." It must be confessed that there is considerable ambiguity at times, which, perhaps, is due to the condensation. There is no mention of hammer and chisel as a means of entering the cranium in place of the trephine. Certainly there is no comparison between them in the matter of speed and ease to the operator, and the theoretical consideration that concussion is thereby added to we have never seen borne out clinically.

The chapter upon Surgical Diseases of the Abdomen and Pelvis is thoroughly modern and the text much enhanced by numerous illustrations. Much valuable information is contained in the few paragraphs upon the method of operating within the abdomen and pelvis.

In discussing the surgery of the heart and blood-vessels we have been given good, sound, surgical advice; the same may be said of diseases and injuries of bones.

Fractures and Dislocations are given in a terse, yet quite complete, way that is certain to please the student. Few surgeons, we imagine, will agree with the author that hooks are better than wiring in patillar fractures. In his own mind there is no question as to the correctness of his opinion. He says: "The treatment of fractured patella by wiring the fragments, and the management of cases with long fibrous union by resection and wiring, are not justifiable. The disability resulting from imperfect connection of the fragments is not great enough to warrant the additional though slight risk assumed. Such, at least, is my opinion. I am prepared to have the fracture in my own person treated by hooks, but not by wiring. Therefore, I recommend one and condemn the other."

In the diagnosis of dislocations of the head of the humerus Dugas's rule is not mentioned,—a misfortune, it seems to us, since it is so easily remembered and so reliable.

Fractures of the lower end of the radius are treated in a very common sense, practical way which will meet the approval of most surgeons.

The book abounds in excellent things, omissions being few and usually of unimportant matters. Both students and practitioners will find it most convenient and satisfactory.

The illustrations are numerous and many of them new, a tribute alike to the originality of the author and the liberality of the publishers. J. P.

